# Helminth Infections of Rodents and Their Zoonotic Importance in Boyer-Ahmad District, Southwestern Iran

**Published:** 2017

**Authors:** Mohammad Javad RANJBAR, Bahador SARKARI, Gholam Reza MOWLAVI, Zeinab SEIFOLLAHI, Abdolali MOSHFE, Samaneh ABDOLAHI KHABISI, Iraj MOBEDI

**Affiliations:** 1. Dept. of Parasitology and Mycology, School of Medicine, Shiraz University of Medical Sciences, Shiraz, Iran; 2. Dept. of Parasitology and Mycology, School of Public Health, Tehran University of Medical Sciences, Tehran, Iran; 3. Cellular and Molecular Research Center, Yasuj University of Medical Sciences, Yasuj, Iran; 4. Dept. of Parasitology and Mycology, School of Medicine, Zahedan University of Medical Sciences, Zahedan, Iran

**Keywords:** Helminthic parasites, Rodents, Iran

## Abstract

**Background:**

Rodents are considered as reservoirs of various zoonotic diseases including helminthic infections. The current study aimed to evaluate the prevalence of helminth infections in rodents, in Boyer-Ahmad district, Southwestern Iran.

**Methods:**

Overall, 52 rodents were captured from various areas of the district by Sherman live traps. The animals were then euthanized and dissected. During necropsy, each organ was examined macroscopically for presence of any cyst or visible parasite. The gastrointestinal tract was removed and their contents were evaluated for larva or adult worms. *Trichinella* larvae in the rodents’ muscles were investigated by both digestion and pathological methods.

**Results:**

Twenty-eight (53.8%) of the trapped rodents were male. The rodents were including 25 (48.1%) *Meriones persicus*, 1(1.9%) *Calomyscus bailwardi*, 1 (1.9%) *Arvicola terresterris*, 7 (13.5%) *Rattus rattus*, 8 (15.4%) *R. norvegicus*, and 10 (19.2%) *Apodemus sylvaticus*. Of them, 38 (73.0%) were infected with at least one helminth. Collected rodents were infected with *Hymenolepis diminuta* (50%), *Hymenolepis nana fraterna* (28.8%), *Skrjabinotaenia* sp. (15.4%), *Anoplocephalidae* sp. (15.4%), *Cysticercus fasciolaris* (5.8%), *Trichuris muris* (36.5%), *Aspiculuris tetraptera* (15.4%), *Syphacia* sp. (5.7%), *Rictularia* sp. (15.4%), *Trichostrongylus* sp. (3.8%), and *Gongylonema* sp. (3.8%). *M. persicus* was the most (84%) infected rodent, yet the differences between rodent genus and helminth infectivity were not statistically significant (*P*>0.05).

**Conclusion:**

The rodents in Boyer-Ahmad district are infected with different helminths infections that some of them are recognized as threat to human health.

## Introduction

Rodents are considered as reservoirs of various zoonotic diseases including parasitic infections (1). Rodent’s fur, saliva, urine, and feces, may contain pathogens, which may contaminate human, or domestic animals (1). Evaluation of the helminthiases of rodents in different zoographical areas seems necessary due to the impact of rodent-associated diseases on human and livestock health.

Several studies have been performed on endoparasites of rodents in different areas of Iran, including Tehran, Khuzestan, Ardabil, Golestan, Kashan, and Hamedan (2–8). Nevertheless, the parasitic fauna of the rodents in each ecological setting might be different because of environmental differences across the country and further studies in areas with different ecological setting seem necessary.

A study on gastrointestinal parasites of rodents in Tabriz revealed various helminth infections in more than 50% of the rodents (9). Among the isolated helminthes, *H. diminuta* and *H. nana* were detected in 22.3% and 4.31% of the rodents, respectively (9).

The helminth infections of *Rattus ratus* and *Rattus norvigicus* were evaluated in Tehran and reported *H. nana fraterna* in 35.8% and *H. diminuta* in 7.5% of the rodents (5). Endoparasites of wild rodents in southeastern Iran were evaluated and found *H. diminuta* in 11% and *H. nana* in 8%, of the rodents (10). Other species of helminthes including *Trichuris* sp., *Skerjabinotaenia*, and *Trichostrongylus* spp., were also detected in the rodents (10). *H. diminuta* was the most prevalent helminth species reported (10). Similarly, *H. diminuta* was the most common parasite that could be found in different species of rodents in Dashte Moghan, in Ardabil Province (4). An infection rate of 31.3 and 12.5% were reported for *H. nana* and *H. diminuta* respectively, amongst wild rodents in Khuzestan, southwest of Iran (11). *H. nana* and *H. diminuta* are common helminths of rodents in Iran. However, infection with these two important zoonotic helminths has not been observed in *Apodemus sylvaticus* in suburban areas of Hamadan City, western Iran (8). In a study on helminth parasites of 77 *Rhombomys opimus* collected from rural areas of Golestan Province, 81.8% of the gerbils were found infected with at least one species of helminths (2). *H. diminuta* is one of the most important zoonotic helminths isolated from rodents in different areas of Iran and nearby countries (8, 10–13). In Gaza strip 36.6% of the trapped rodents were infected with *H. diminuta.* Another study on helminth infections of rodents in Doha, Qatar, revealed that 35.8% of *R. norvigicus* captured in urban areas of the country were infected with *H. diminuta.*

The current study was conducted to find out the helminth infections in rodents, in Boyer-Ahmad district, Southwestern Iran.

## Materials and Methods

### The Study Area

Boyer-Ahmad district is the main Township of the Kohgiluyeh and Boyer-Ahmad Province, in southwest Iran with a tropical and a cold climate. The warm regions consist of Gachsaran and Kohgiluyeh, while the cold regions include Boyer-Ahmad and Dena. Most areas of the district are overspread with oak trees, wild pistachio, and mountain almond. Local residents mainly live in agricultural practice and animal husbandry. Wildlife of the region are including brown bear, types of eagles, leopards, wolves, many species of wild cats, falcons, and partridges.

### Rodents’ Collection and Identification

After getting approval from the Ethics Committee of the institute (SUMS), 52 rodents were collected during Jun to Nov 2014, using Sherman live traps with roasted almonds, as bait. The traps were placed around the rodent nest late afternoon and were collected next early morning. Different areas of the district were selected for sampling. All trapped rodents were transferred to animal laboratory of Shiraz University of Medical Sciences and were identified based on morphological characteristics.

Rodents were anesthetized and blood samples were collected from their heart. The animals then were euthanized and dissected. During necropsy, each organ was examined macroscopically for presence of any cyst or visible parasite. The lung and abdominal viscera including spleen, liver, gallbladder, heart, kidney and urinary bladder were examined for the presence of any cyst or helminths. Brain tissues were also examined by means of impression smear. The gastrointestinal tract including stomach and small and large intestine were removed. The intestinal contents were transferred to large Petri dishes, containing saline solution, and larva or adult worms were collected under stereomicroscope. Moreover, mucosa of digestive tract was carefully examined for any adult worm or helminths’ larvae.

The helminths were isolated, counted and preserved in buffered 10% formalin and 5% glycerin alcohol. Isolated helminths were cleared with lactophenol and stained with FAAL (Formalin Azocarmine Alcohol ethylic and Lactic acid) and carmine staining solutions and then were mounted, using Canada balsam. Identification of helminths was done, using appropriate systematic keys (14). *Trichinella* larvae in the rodents’ muscles were investigated by both digestion and pathological methods. For pathological investigation, sections were prepared from the muscles and stained with Hematoxylin eosin (HE). Stool samples of rodents were examined with the Telemann method.

### Statistical Analysis

The statistical analysis was done using SPSS software (ver. 18, Chicago, IL, USA). Chi-square test was used to examine the association of rodent’s parasitic infections in different rodent groups.

## Results

Twenty-eight of rodents (53.8%) were males. Mean weight of the rodents was 117.79 gr (ranged from 45–300 gr, SD=87.23) and most of them (55.8%) were in weight group of 51–100 gr, while a few (13.5%) were relatively big and heavy (>250 gr).

Trapped rodents were including 25 (48.1%) *M. persicus,* 1(1.9%) *Calomyscus bailwardi,* 1 (1.9%) *Arvicola terresterris,* 7 (13.5%) *R. rattus*, 8 (15.4%) *R. norvegicus*, and 10 (19.2%) *Apodemus sylvaticus*. Out of 52 collected rodents, 38 (73%) were infected with at least one helminth while 14 (27.0%) of them were non-infected. The most infected (84.0%) rodent was *M. persicus.*

**Fig. 1:**
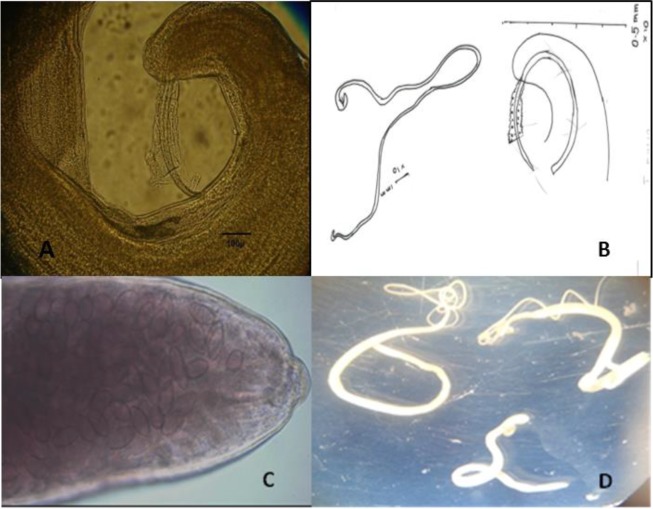
*Trichuris muris* isolated from large intestine of rodents. A: posterior end of male (20×) showing spicule with sheath; B: *T. muris,* male drawn by camera lucida; C: *T. muris* posterior end of female (20×); D: Adult worm

**Fig. 2:**
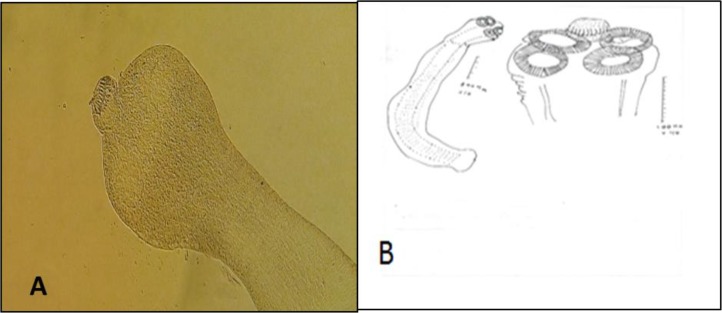
*H. nana* isolated from the rodents’ small intestine. A: Scolex (10×); B: Scolex and adult worm drawn by camera lucida

**Fig. 3:**
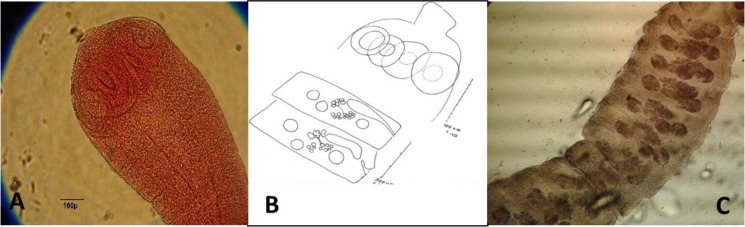
*H. diminuta* isolated from the rodents’ small intestine. A: Scolex (20×); B: Scolex and mature segment drawn by camera lucida; C: Mature segment showing ovary and testes (10×)

**Fig. 4:**
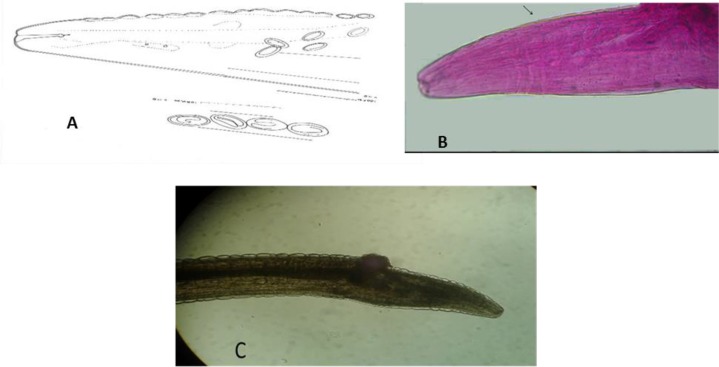
*Gongylonema* sp. isolated from rodents stomach A: Anterior region drawn by camera lucida (20×); B: Anterior region with Carmen staining (10×); C: Anterior region without staining

**Fig. 5:**
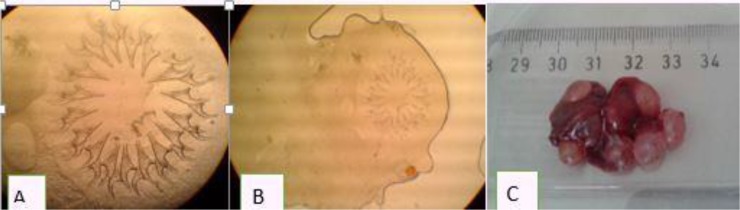
*Cysticercus fasciolaris.* A: Hooks; B: Scolex; C: Infected liver

**Fig 6:**
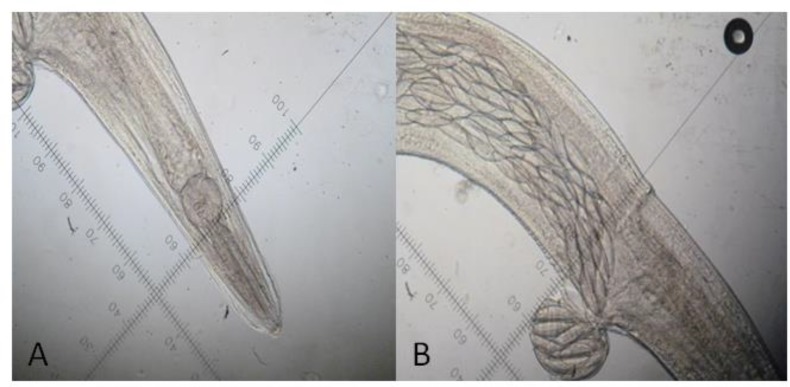
*Syphacia* sp. isolated from rodents large intestine. A: Anterior region showing prominent lip and esophagus bulb (10×); B: Vulva opening with egg (20×)

**Fig. 7:**
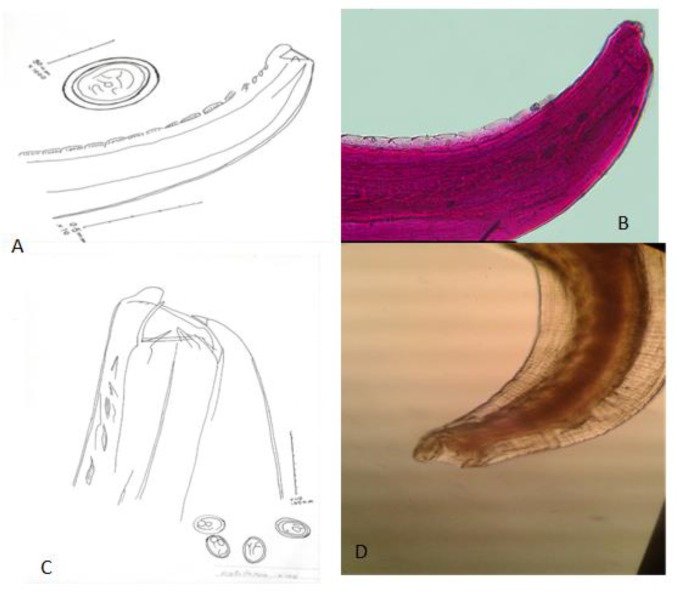
*Rictularia* sp. A: Anterior region and egg (camera lucida drawing); B: Anterior region showing sub ventral comb-like spine (10X); C: Anterior region showing teeth and egg; D: Anterior region showing glandular and muscular esophagus

Collected rodents were infected with *H. diminuta* (50.0%), *H. nana fraterna* (28.8%), *Skrjabinotaenia* sp. (15.4%), *Anoplocephalidae* sp. (15.4%), *Cysticercus fasciolaris* (5.8%), *Trichuris muris* (36.5%), *Aspiculuris tetraptera* (15.4%), *Syphacia* sp. (5.8%), *Rictularia* sp. (15.4%), *Trichostrongylus* sp. (3.8%), and *Gongylonema* sp. (3.8%). Figs. 1– 7 show some of the detected helminths in the studied rodents.

*M. persicus* was the most (84.0%) infected rodent, yet the differences between rodent’s genus and helminth infectivity were not statistically significant (*P*>0.05). Likewise, there was no association between helminth infectivity and sex or weight of the rodents.

Table 1 shows the helminth infection of captured rodents based on the rodent species. No helminth infection was detected in the lung, brain, blood or bladder of the studied animals. No *Trichinella* larvae were detected in any of studied rodents when evaluated by either pathological or tissue digestion methods.

**Table 1:** Helminthic infection of collected rodents in Boyer-Ahmad district, southwestern Iran, based on rodent species

***Rodent Infection***	***Rattus rattus (N=7)***		***Rattus norvegicus (N=8)***		***Meriones persicus (N=25)***		***Calomyscus bailwardi (N=1)***		***Apodemus sylvaticus (N=10)***		***Arvicola terresterris (N=1)***		***Total (N=52)***	
	**No.**	**%**	**No.**	**%**	**No.**	**%**	**No.**	**%**	**No.**	**%**	**No.**	**%**	**No.**	**%**
*Trichuris muris*	0	0	0	0	16	64	0	0	3	30	0	0	19	36.53
*Syphacia* sp.	0	0	0	0	2	8	0	0	0	0	1	100	3	5.76
*Aspiculuris tetraptera*	0	0	0	0	5	20	0	0	3	30	0	0	8	15.38
*Gongylonema* sp.	0	0	2	25	0	0	0	0	0	0	0	0	2	3.84
*Rictularia* sp.	0	0	0	0	8	32	0	0	0	0	0	0	8	15.38
*Trichostrongylus* sp.	0	0	0	0	0	0	0	0	2	20	0	0	2	3.84
*Hymenolepis diminuta*	5	71.4	2	25	17	68	0	0	2	20	0	0	26	46
*Hymenolepis nana*	2	28.57	0	0	11	44	0	0	2	20	0	0	15	28.8
*Anoplocephalidae sp.*	5	71.4	1	12.5	2	8	0	0	0	0	0	0	8	15.38
*Skrjabinotaenia* sp.	3	42.9	0	0	5	20	0	0	0	0	0	0	8	15.38
*Cysticercus fasciolaris*	0	0	2	25	0	0	0	0	1	10	0	0	3	5.76

## Discussion

Rodents are recognized as a reservoir of different zoonotic diseases including parasitic diseases and pose a serious threat to human health (1). Rat associated health risk is significant in areas where people are in close contact with these animals (1). The prevalence of parasitic infection in rat population is highly variable in different geographical areas. Determination of parasitic infection of rats is important for accurately measuring the presence, magnitude, and nature of rat-associated health threats in any zoogeographical areas and to reduce and prevent transmission of rat-to-human diseases (1).

The present study gives an overview of the helminth infections of trapped rodents in Boyer-Ahmad district, southwestern Iran. Eleven helminth species were detected from six species of rodents. A few of detected helminths from the rodents, including *H. nana* and *H. diminuta* were detected in human in previous studies in Iran (12). *H. nana* and *H. diminuta,* which are prevalent in rats, are potentially transmissible to human. *H. nana* is a common infection in children in Iran while sporadic cases of *H. diminuta* have been reported from Iran or other areas in the world (12–13, 15). In a recent study, *H. nana* has been detected in children in the same studied area (16).

The rate of infection with *H. dimninuta* in the current study (50%) is somehow similar to the results of the study conducted on rodents in Germi, Dasht Moghan in Ardabil province reporting *H. diminuta* in 38.8% of the rodents (4). The rate of infection with this helminth is also somewhat similar to the rate reported for this helminth in rodents from Gaza strip, Palestine (36.6%) and Doha, Qatar (35.8%) (12–13). Lower infection (12.3%) of rodent with *H. diminuta* has been reported in Kermanshah, in the western part of Iran (6).

In the current study, statistical analysis revealed no significant differences between sex of rodents and infectivity with different helminths. Such associations were found between sex and infectivity with *H. nana* and *T. muris,* where females were more infected than males (2).

Climate, season and weather play significant roles in the ecology of rat-associated zoonoses through their effect on the biology and ecology of rodents (1). Understanding which rats pose the greatest health risk for human is important. In the current study, the most infected rodent was *M. persicus.* In accordance with this finding, a study in Ardabil Province revealed that *M. persicus* were more infected than other species of rodents, namely *M. socialis* (4).

*Cysticercus fasciolaris*, the larval stage of *Taenia taenioformis* was detected in 5.76% of the studied rodents in the present study. This cestode is a common helminthic infection of rodents and has been reported in previous studies in Iran (4, 6, 11). Other species of *Cysticercus* including *Cysticercus tenuicollis,* the larval stage of *Taenia hydatigena,* has been isolated from the wild boars is southwestern of Iran (17).

Findings of the current study highlighted the major parasitic infection of rodent in Boyer-Ahmad district and revealed that the rodents in Kohgiluyeh and Boyer-Ahmad province are infected with different helminths infections that some of them are recognized as threat to human health. These rodents contaminate the environment, food, plants and water sources and pose a health threat to local residents and domestic animals. These animals may act as reservoir of leishmaniasis, which is uncommon in the studied area (18).

Helminthic infections such as cystic echinococcosis and fascioliasis are quite common in this province (19–21). Rodents are currently considered as a reservoir of fascioliasis in some areas of the world (22,23). Moreover, rodents are considered to be infected with a few of zoonotic protozoan, which have previously been reported in this province (24, 25). Wide-ranging surveys with more samples in different seasons of the year can be suggested to determine the possible infection of rodents with *Fasciola* sp. in this area.

## Conclusion

Intestinal infection of rodents with helminths can be caused by using of animal and human dung and insanitary burial of domestic garbage. In order to clarify the epidemiological condition of infected rodents, comprehensive studies should be performed in other cities of the province.
